# A Systematic Review and Meta-Analysis of the Association between Serotonergic Gene Polymorphisms and Obstructive Sleep Apnea Syndrome

**DOI:** 10.1371/journal.pone.0086460

**Published:** 2014-01-27

**Authors:** Huajun Xu, Jian Guan, Hongliang Yi, Shankai Yin

**Affiliations:** Department of Otolaryngology, Shanghai Jiao Tong University Affiliated Sixth People’s Hospital, Otolaryngology Institute of Shanghai Jiao Tong University, Shanghai, China; National Institute for Viral Disease Control and Prevention, CDC, China, China

## Abstract

**Background:**

5-Hydroxytryptamine receptor (5-HTR) and 5-hydroxytryptamine transporter (5-HTT) gene polymorphisms have been reported to be associated with susceptibility to obstructive sleep apnea syndrome (OSAS). The associations, derived from sporadic, inconsistent, small-sample-size studies, need to be evaluated further in a meta-analysis.

**Methods:**

Relevant studies were identified by searching PubMed, Embase, The Cochrane Library, China National Knowledge Infrastructure (CNKI), Wanfang, and Weipu. Eligible data were extracted from each included study. Odds ratios (ORs) were calculated using a fixed-effects or a random-effects model. The ORs and 95% confidence interval (CI) were used to assess the strength of the association between serotonergic gene polymorphisms and OSAS in the dominant and recessive models, as well as alleles. The Q statistic was used to evaluate homogeneity and Begg’s test was used to assess publication bias.

**Results:**

Eight studies were finally included in the meta-analysis of the association between 5-HTR2A gene variants (including 102T/C and 1438G/A), 5-HTT gene polymorphisms (including 5-HTT gene-linked promoter region (5-HTTLRP), and serotonin transporter intron 2 variable number tandem repeat (STin2VNTR) and OSAS risk. The G allele of 5-HTR2A 1438G/A, long 5-HTTLPR, and 10-tandem-repeats STin2VNTR were shown to increase OSAS susceptibility, with ORs of 2.33 (A vs. G, 95% CI 1.48–3.66), 1.24 (L vs. S, 95% CI: 1.04–1.49), and 2.87 (10 vs. 12, 95% CI: 1.38–5.97), respectively. These significant differences were determined in both dominant and recessive models. Of the 5-HTR2A 1438G/A gene polymorphism, the AA genotype increased the OSAS risk, with an OR of 4.21 (95% CI: 2.83–6.25) in a recessive model in male OSAS patients, but no significant association was found in females.

**Conclusions:**

Our meta-analysis demonstrated that polymorphisms in the 5-HTR2A 1438G/A and 5-HTT genes contributed to susceptibility to OSAS. The A allele of the 1438G/A gene polymorphism is predominantly distributed in males and increased the OSAS risk significantly.

## Introduction

Because of its high morbidity and mortality, obstructive sleep apnea syndrome (OSAS) has recently become an important public health issue. It affects about 2% of females and 4% of males in middle-aged groups in Western counties [1]. Repeated partial or complete collapse of the pharynx during sleep, which results in apnea or hypopnea associated with oxygen desaturation and arousal from sleep, is the most characteristic feature of OSAS [2]. The pathophysiology of OSAS is associated with activation of a number of neural, humoral, thrombotic, metabolic, and inflammatory disease mechanisms [3].

Serotonergic neurons innervating motoneurons increase their firing rates in response to respiratory challenges, and long-term facilitation of respiratory activity in response to hypoxia is 5-hydroxytryptamine (5-HT, serotonin)-dependent [4]. Thus, it is reasonable that the receptor and transporter of 5-HT are also involved in the pathophysiological process of OSAS.

There is a large family of 5-HT receptors [5]. Among them, 5-HT receptor (5-HTR) 2A/2C has been most studied. The 5-HTR 2A gene is located on chromosome 13q14–q21 and the 5-HTR 2C gene is located in the q24 region of chromosome X [6]–[7]. Binding with the 2A/2C receptors, 5-HT plays a vital role in preventing glossocoma and sustaining patency of the upper airway [8]. A series of case control studies reported an association between 5-HTR 2A/2C gene polymorphisms and OSAS [9]–[14]. In 5-HTR 2A, two polymorphisms have been reported: a 1438A/G polymorphism in the promoter region and an MspI polymorphic site at position 102T/C [12]. A 796C/G polymorphism locus in the 5-HTR 2C gene has also been identified [13].

The 5-HT transporter (5-HTT, serotonin transporter) gene is located in the 17q11.1–17q12 region of chromosome 17 [15]. Two gene polymorphisms, the 5-HTT gene-linked polymorphic region (5-HTTLPR) and the intron 2 variable numbers of tandem repeats (STin2VNTR), have been studied in connection with OSAS susceptibility because STin2VNTR can affect enhancer function and transcription of the 5-HTT gene and 5-HTTLPR can influence 5-HT reuptake [16]–[18].

Although the various studies have provided important serotonergic genetic information regarding susceptibility to OSAS [9]–[14], [19], [20], because of the limitations of small sample sizes in each study, low statistical power, and inconsistent results, they are incapable of demonstrating a reliable association. Also, the associations hinted at from sporadic cases and populations of differing ethnic origins need to be re-analyzed comprehensively. In this report, to further evaluate the associations between 5-HT receptor and transporter gene polymorphisms and the risk of OSAS, we performed a meta-analysis by combining the genotyping data from eligible studies published to date.

## Materials and Methods

This study was strictly followed the guidelines of the PRISMA (Preferred Reporting Items for Systematic Reviews and Meta-Analysis).

### Publication Search

To identify all eligible studies that address the association between 5-HT receptor and transporter gene polymorphisms and OSAS, we conducted key word searches in the biomedical electronic databases, including PubMed, Embase, The Cochrane Library, China National Knowledge Infrastructure (CNKI), Wanfang, and Weipu: (obstructive sleep apnea hypopnea syndrome or OSAHS or obstructive sleep apnea syndrome or OSAS or obstructive sleep apnea or OSA or sleep apnea or apnea) and (5-hydroxytryptamine receptor or 5-HTR or serotonin receptor or 5-hydroxytryptamine transporter or 5-HTT or serotonin transporter) in combination with (gene or polymorphism or variants or alleles) were used as research terms. The final search was updated in June 2013. The search was limited to studies published in English and Chinese.

### Inclusion and Exclusion Criteria

Case-control studies that met the following criteria were included: 1) published in peer-reviewed journals, 2) contained independent data, 3) had enough data to evaluate the relationship between gene polymorphisms and OSAS susceptibility, and 4) had enough data to determine the genotype distributions in both the case and control groups. The authors were contacted if there were queries regarding the studies.

Studies were excluded for the following reasons: 1) lack of a suitable control group, 2) no usable data reported, 3) data were contradictory, 4) repeated data, and 5) abstracts or reviews.

### Data Extraction

Two investigators (Drs. Xu and Guan) performed the data search, data screening, and data extraction independently. Disagreements regarding data screening and extraction were resolved by discussion. If there was still disagreement, a third reviewer (Prof. Yin) participated to resolve the issue. The following data were extracted from the original studies: first author, year of publication, country, sample size, age, gender, body mass index (BMI), apnea-hypopnea index (AHI), and genotype frequency.

### Statistical Methods

The Review Manager software (ver. 5.0, Cochrane Collaboration, Oxford, UK), the SAS software (ver. 9.1.3, SAS Institute Inc., Cary, NC), and Stata (ver. 11.0, Stata Corporation, College Station, TX) were used for the meta-analysis and statistical analyses. For genetic frequency distributions, Hardy-Weinberg equilibrium (HWE) was calculated with the SAS software to assess goodness of fit. Homogeneity was tested using Cochran chi-squared tests. The Mantel-Haenszel fixed-effects model or the random-effects model was used for the meta-analysis in the comparison of serotonergic gene polymorphisms [21]–[22]. Begg’s test was used to evaluate publication bias.

## Results

### Search Results

**Figure 1:**
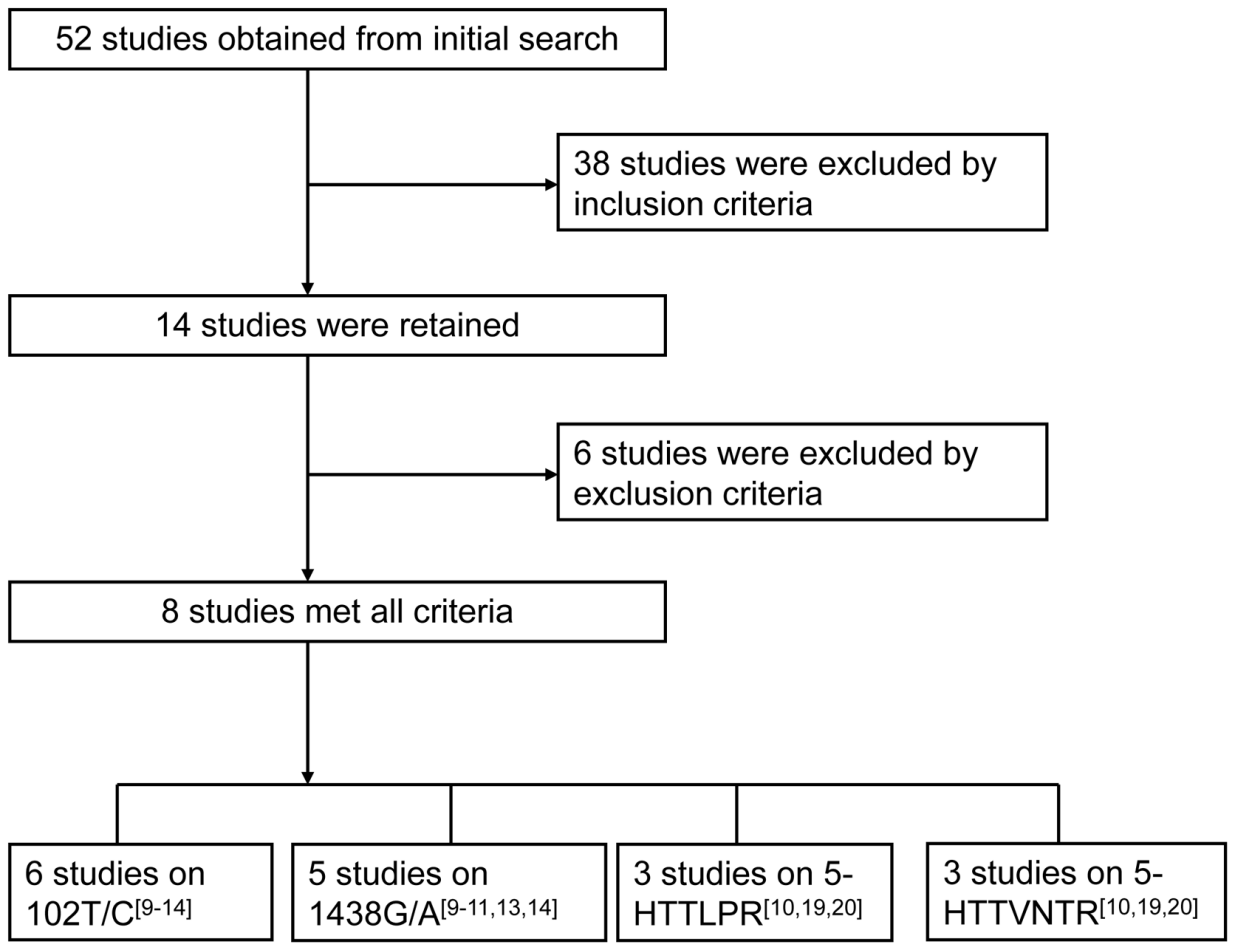
Flow chart of literature search and study selection. 6, 5, 3 and 3 studies were eligibly included in the meta-analysis of 5-HTR2A 102 T/C, 5-HTR2A 1438 G/A, HTTLRP and HTTVNTR, respectively.

In total, 52 studies were identified by electronic and manual searching. At the stage of screening by the inclusion criteria, 38 studies were excluded for the following reasons: animal studies, cell studies, reviews, or not correlated with OSAS. At the stage of screening by the exclusion criteria, after reading the full articles, six studies were excluded for the following reasons: two articles were repeated studies [23]–[24], one was an abstract [25], two articles lacked complete data [26]–[27], and one article had contradictory data [28]. Finally, eight studies met the exclusion criteria and were included in our systemic review and meta-analysis (Figure 1). Of the eight studies, six had data for 5-HTR2A 102 T/C, five for 5-HTR2A 1438 G/A, three for 5-HTTLRP, and three for STin2VNTR (Figure 1, Table S1).

### Study Characteristics

All eligible studies were published between 2005 and 2013. Five studies were from China [10], [13], [14], [19], [20], one from Turkey [9], one from Brazil [11], and one from Japan [12] (Table S1). The studied populations were mainly middle-aged and slightly overweight in cases and controls, although one study lacked this information (Table S1). One study did not provide gender data [12]; the male proportion in the remaining populations ranged from 40.0% to 88.6% (Table S1). The BMI ranged from 21.3±7.5 kg/m^2^ to 29.3±3.7 kg/m^2^, although one study lacked these data [9]. The AHI of OSAS populations ranged from ≥5 to 53.9±16.4 times per h; the AHI of control groups were all below 5 (Table S1). All polymorphisms were identified by polymerase chain reaction (PCR) or PCR-restriction fragment length polymorphism (PCR-RFLP) techniques.

The allele frequencies of all OSAS and control samples were in HWE except one study (Table 1). The combined samples for 5-HT2A 102C/T [9]–[14], 5-HT2A 1438G/A [9]–[11], [13], [14], 5-HTTLPR [10], [19], [20], and STin2VNTR [10], [19], [20] polymorphisms for the meta-analysis in case and control groups were 566, 466, 593, and 593 and 728, 551, 479, and 479, respectively (Table S1).

**Table 1:** Genotypes and alleles distribution in OSAS and control groups.

	Genotypes						Alleles				HWE
Study	OSAS			Control			OSAS		Control		
**102C/T**	**TT**	**TC**	**CC**	**TT**	**TC**	**CC**	**T**	**C**	**T**	**C**	
**Bayazit Y, 2006** [**9**]	17	21	17	28	54	20	55	55	110	94	Yes
**Chen H, 2013** [**10**]	34	56	31	29	54	22	124	118	112	98	Yes
**De Carvalho T, 2013** [**11**]	23	66	11	20	69	11	112	88	109	91	No
**Sakai K, 2005** [**12**]	47	90	40	25	46	29	184	170	96	104	Yes
**Yin G, 2012** [**13**]	52	107	51	30	51	24	NM	NM	NM	NM	Yes
**Zhu J, 2007** [**14**]	18	30	17	15	28	11	66	64	58	50	Yes
**1438G/A**	**AA**	**AG**	**GG**	**AA**	**AG**	**GG**	**A**	**G**	**A**	**G**	
**Bayazit Y, 2006** [**9**]	24	19	12	25	50	27	67	43	100	104	Yes
**Chen H, 2013** [**10**]	74	30	17	28	28	49	178	64	84	126	Yes
**De Carvalho T, 2013** [**11**]	35	61	4	19	71	10	131	69	109	91	No
**Yin G, 2012** [**13**]	85	82	43	17	67	21	252	168	101	109	Yes
**Zhu J, 2007** [**14**]	41	15	9	15	14	25	97	33	44	64	Yes
**5-HTTLPR**	**SS**	**SL**	**LL**	**SS**	**SL**	**LL**	**S**	**L**	**S**	**L**	
**Chen H, 2013** [**10**]	59	35	27	55	34	16	158	84	146	64	Yes
**Yue W, 2005** [**20**]	51	33	20	78	54	18	135	73	210	90	Yes
**Yue W, 2008** [**19**]	114	106	34	173	131	34	334	174	477	199	Yes
**STin2VNTR**	**10/10**	**10/12**	**12/12**	**10/10**	**10/12**	**12/12**	**10**	**12**	**10**	**12**	
**Chen H, 2013** [**10**]	8	24	89	1	10	94	64	178	12	198	Yes
**Yue W, 2005** [**10**]	3	20	81	1	14	135	26	182	16	284	Yes
**Yue W, 2008** [**19**]	6	46	202	3	41	294	58	450	47	629	Yes

Abbreviation: OSAS, obstructive sleep apnea syndrome; NM, not mentioned; S/L allele, short/long allele; 5-HTTLPR, 5-HTT gene-linked polymorphic region; STin2VNTR, serotonin transporter intron 2 variable number tandem repeat; HWE, Hardy–Weinberg equilibrium.

### Meta-analysis of the Association between 5-HTR and 5-HTT Gene Polymorphisms and OSAS

The association between gene polymorphisms and susceptibility to OSAS was evaluated in the total case and control populations combined from eligible studies of each gene locus. All genetic loci were tested in a dominant model, a recessive model, and a susceptibility allele versus insusceptibility allele model.

**Figure 2:**
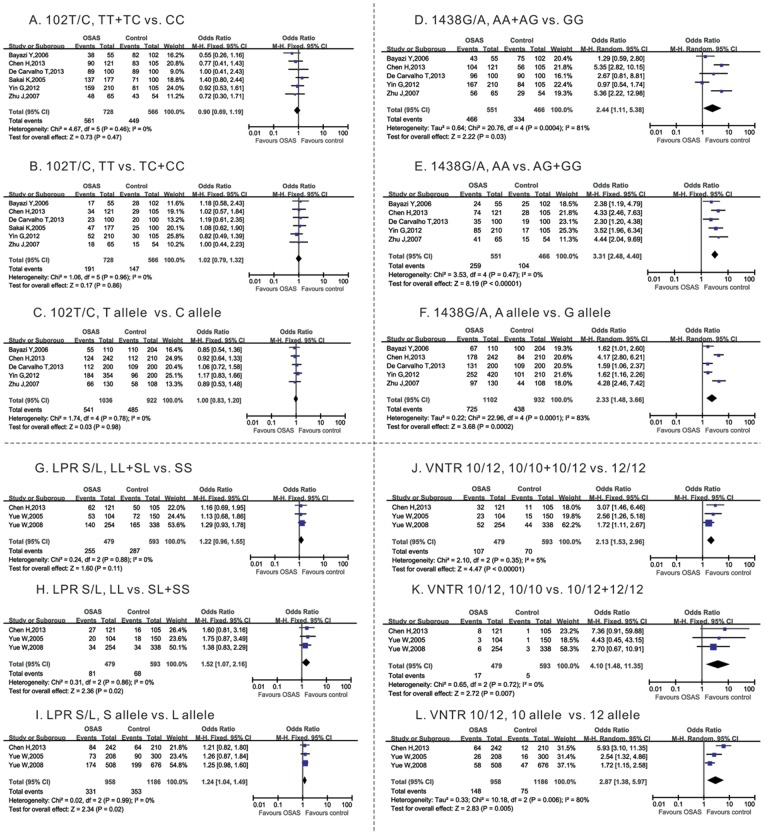
Forest plots for the associations between 5-HTR2A 102 T/C, 5-HTR2A 1438 G/A, HTTLRP and HTTVNTR gene polymorphisms and OSAS risk. The squares correspond to the study specific OR and 95% CI. The area of the squares reflects the weight. The diamond represents the summary OR and 95% CI.

Of the 5-HTR2A 102T/C polymorphisms, six studies containing 566 case subjects and 728 controls were included in this meta-analysis [9]–[14]. The I^2^ of the dominant models (TT+TC vs. CC), recessive model (TT vs. TC+CC) and T allele vs. C allele were 0 and the p values of the Q test were higher than 0.1, suggesting no significant heterogeneity among the studies, so we used a fixed-effects model to analyze the data. As shown in Table 2, meta-analysis of the dominant model (TT +TC vs. CC), recessive model (TT vs. TC +CC), and T allele vs. C allele revealed that no genotype or allele was associated with OSAS risk (Table 2, Figure 2A, B, C).

**Table 2:** Meta-analysis of the association between gene polymorphisms and OSAS risk.

	Genotypes	No.	Test of association			Test of heterogeneity			
Polymorphism			OR	95% CI	P value	Model	Q	P value	I^2^
**102T/C**	TT+TC vs.CC	6	0.90	0.69–1.19	0.47	F	4.67	0.46	0
	TT vs. TC+CC	6	1.02	0.79–1.32	0.86	F	1.06	0.96	0
	T allele vs. C allele	5	1.00	0.83–1.20	0.98	F	1.74	0.78	0
**1438G/A**	AA+AG vs. GG	5	2.44	1.11–5.38	0.03	R	20.76	0.47	81
	AA vs. AG+GG	5	3.31	2.48–4.40	<0.01	F	3.53	<0.01	0
	A allele vs. G allele	5	2.33	1.48–3.66	<0.01	R	22.96	<0.01	83
**5-HTTLPR L/S**	LL+SL vs. SS	3	1.22	0.96–1.55	0.11	F	0.24	0.86	0
	LL vs. SL+SS	3	1.52	1.07–2.16	0.02	F	0.31	0.88	0
	L allele vs. S allele	3	1.24	1.04–1.49	0.02	F	0.02	0.99	0
**STin2VNTR 10/12**	10/10+10/12 vs.12/12	3	2.13	1.53–2.96	<0.01	F	2.1	0.35	5
	10/10 vs.10/12+12/12	3	4.10	1.48–11.35	<0.01	F	0.65	0.72	0
	10 allele vs. 12 allele	3	2.87	1.38–5.97	<0.01	R	10.18	<0.01	80

Abbreviation: L or S, long or short 5-HTT gene-linked polymorphic region;10 or 12, serotonin transporter intron 2 variable number tandem repeat; F, fixed-effects model; R, random-effects model; OR, odds ratios; 95%CI, 95% confidence interval.

For the 5-HTR2A 1438G/A polymorphism, five studies had 466 case subjects and 551 controls [9]–[11], [13], [14]. The I^2^ of the dominant models (AA+AG vs. GG) and the A allele vs. G allele were over 50%, and the p values of the Q test were 0.47 and <0.01, respectively, suggesting strong heterogeneity among the studies. So, these data were analyzed using a random-effects model. The meta-analysis revealed that the A allele was associated with OSAS risk with an OR of 2.44 (95% CI: 1.11–5.38) in the dominant model (AA+AG vs. GG). The A allele increased the OSAS risk with an OR of 2.33 (95% CI: 1.48–3.66) compared with the G allele (Table 2, Figure 2D, F). The I^2^ of the recessive model (AA vs. AG+GG) was 0 and the p value of the Q test was less than 0.01, so we used a fixed-effects model to analyze the data. As shown in Table 2 and Figure 2E, the A allele was associated with OSAS risk with an OR of 3.31 (95% CI: 2.48–4.40) in the recessive model (AA vs. AG+GG).

Of the long or short 5-HTTLPR, three studies were pooled for totals of 593 and 479 subjects in the OSAS and control groups [10], [19], [20], respectively. The I^2^ of the dominant model (LL+SL vs. SS), recessive model (LL vs. SL+SS), and L allele vs. S allele were 0 and p values of the Q test were all more than 0.1. Meta-analysis with a fixed-effects model showed that the LL genotype increased the OSAS risk at an OR of 1.52 (95% CI: 1.07–2.16) when compared with a population with LS or SS genotypes. A single allele L increased the OSAS risk at an OR of 1.24 (95% CI: 1.04–1.49) compared with the allele S (Table 2, Figure 2G and I). The dominant model (LL+SL vs. SS) showed no significant association with OSAS risk (Table 2, Figure 2H).

For the STin2VNTR gene polymorphism, three studies were pooled with 593 and 479 subjects in the OSAS and control groups, respectively [10], [19], [20]. The I^2^ of the dominant model (10/10+10/12 vs. 12/12) and recessive model (10/10 vs. 10/12+12/12), were 5 and 0, and the p values of the Q test were 0.35 and 0.72, respectively. Meta-analysis using a fixed-effects model showed that 10 tandem repeats increased the OSAS risk with an OR of 2.13 (95% CI: 1.53–2.96) and 4.10 (95% CI: 1.48–11.35) in a dominant model (10/10+10/12 vs. 12/12) and recessive model (10/10 vs. 10/12+12/12), respectively (Table 2, Figure 2J, K). The 10 tandem repeats variant increased the OSAS risk at an OR of 2.87 (95% CI: 1.38–5.97) compared with 12 tandem repeats (Table 2, Figure 2L).

### Genotype Distribution of 5-HTR 2A and 5-HTT Gene Polymorphisms Sorted by BMI, AHI and Gender

**Figure 3:**
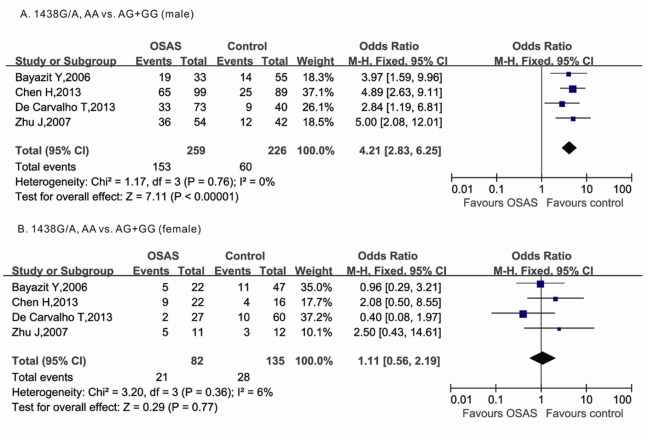
Forest plots for the association between 5-HTR2A 1438 G/A and OSAS risk in male and female groups. The squares correspond to the study specific OR and 95% CI. The area of the squares reflects the weight. The diamond represents the summary OR and 95% CI.

To further evaluate the association between 5-HTR 2A and 5-HTT gene polymorphisms and OSAS susceptibility, we sorted the available data according to BMI, AHI, and gender. The genotype distributions appeared equal within groups categorized by BMI and AHI indexes, consistent with previous reports (data not shown). For gender, two [9], [11], four [9]–[11], [14], one [19], and one [19] studies addressed the 5-HTR2A 102C/T, 5-HTR2A 1438G/A, 5-HTTLPR L/S, and STin2VNTR 10/12 polymorphisms. The genotype distribution of 5-HTR2A 102C/T, 5-HTTLPR L/S, and STin2VNTR 10/12 polymorphisms showed no significant difference between males and females (Table 3), while the AA genotype of 5-HTR 2A 1438G/A in males was significantly more common than in females. The AA genotype increased the OSAS risk with an OR of 4.21 (95% CI: 2.83–6.25) in a recessive model in male OSAS patients; however no significant association was found in females (Figure 3, Table 3).

**Table 3:** Genotype distribution of 5-HTR 2A and 5-HTT gene polymorphisms in OSAS patients subgrouped by gender.

Study	Male		Female		Chi-square with Yates’ correction	Two-tailed p value
**102C/T**	**CC**	**TT+TC**	**CC**	**TT+TC**		
**Bayazit Y, 2006 ** [**9**]	8	25	9	13		
**De Carvalho T, 2013 ** [**11**]	9	64	2	25		
**Pooled**	17 (16%)	89 (84%)	11 (22.4%)	38 (77.6%)	0.548	0.459
**1438G/A**	**AA**	**GG+GA**	**AA**	**GG+GA**		
**Bayazit Y, 2006 ** [**9**]	19	14	5	17		
**Chen H,2013 ** [**10**]	65	34	9	13		
**De Carvalho T, 2013 ** [**11**]	33	40	2	25		
**Zhu J,2007 ** [**14**]	36	18	5	6		
**Pooled**	153 (59.1%)	106 (40.9%)	21 (25.6%)	61 (74.4%)	25.586	<0.0001
**5-HTTLPR L/S**	**LL**	**SS+SL**	**LL**	**SS+SL**		
**Yue W, 2008 ** [**19**]	30 (13.6%)	190 (86.4%)	4 (11.8%)	30 (88.2%)	0.001	0.978
**STin2VNTR 10/12**	**10/10**	**12/12+10/12**	**10/10**	**12/12+10/12**		
**Yue W, 2008 ** [**19**]	4 (1.8%)	216 (98.2%)	2 (5.9%)	32 (94.1%)	0.715	0.398

Abbreviation: 5-HTTLPR L/S, L or S, long or short 5-HTT gene-linked polymorphic region; STin2VNTR 10/12, 10 or 12, serotonin transporter intron 2 variable number tandem repeat. The genotype distribution was expressed as frequency or frequency with percentage.

### Publication Bias

Begg’s test was used to evaluate publication bias in the literature. The shapes of the funnel plots revealed no obvious asymmetry (not shown). We then used Begg’s test to assess statistical evidence of funnel plot symmetry, but no significant publication bias was indicated (Table 4).

**Table 4:** Tests for publication bias in overall population.

Polymorphism	Comparision	Bgger’s test (p value)
**102T/C**	TT+TC vs.CC	0.71
	TT vs. TC+CC	0.71
	T allele vs. C allele	0.73
**1438G/A**	AA+AG vs. GG	0.81
	AA vs. AG+GG	0.81
	A allele vs. G allele	0.81
**5-HTTLPR L/S**	LL+SL vs. SS	1.00
	LL vs. SL+SS	1.00
	L allele vs. S allele	1.00
**STin2VNTR 10/12**	10/10+10/12 vs.12/12	1.00
	10/10 vs.10/12+12/12	1.00
	10/10 allele vs. 12/12 allele	1.00

Abbreviation: 5-HTTLPR L/S, long or short 5-HTT gene-linked polymorphic region; STin2VNTR 10/12, 10 or 12 tandem repeats of serotonin transporter intron 2 variable number tandem repeat.

## Discussion

Although the genetic basis of OSAS is poorly understood, studies to date have focused on relationships between gene polymorphisms and susceptibility to OSAS [9]–[14], [19], [20], [29]. Among genetic variants, polymorphisms in 5-HTR and 5-HTT have been investigated most frequently. Thus, the associations suggested from sporadic, inconsistent, small-sample-size studies needed to be further evaluated in a systematic review and meta-analysis. In this report, the combined number of samples for 5-HT2A 102C/T, 5-HT2A 1438G/A, 5HTTLPR, and STin2VNTR polymorphisms in cases and controls were 566, 466, 593, and 593 and 728, 551, 479, and 479, respectively. Our results revealed that the A allele of 5-HT2A 1438G/A, the long 5-HTTLPR, and 10 tandem repeats of STin2VNTR increased the OSAS risk and that 5-HT2A 102T/C was not associated with OSAS. All risk alleles displayed increased susceptibility to OSAS in both dominant and recessive models except the long 5-HTTLPR, which displayed no significant association with OSAS risk in a dominant model (LL+SL vs. SS); this may be explained by an insufficient sample size or unknown factors. Of the 5-HTR2A 1438G/A polymorphism, the AA genotype increased the OSAS risk with an OR of 4.21 (95% CI: 2.83–6.25) in a recessive model in male OSAS patients; however no significant association was found in the female population. Other gene polymorphisms, such as 5-HTR2A 1421C/T, 1678A/C, 24563A/C, 61472A/C, and 5-HTR2C 796C/G, failed in the meta-analysis due to the limitations of eligible studies or insufficient data. We attempted to categorize the data according to BMI, AHI, and gender to further evaluate the association between gene polymorphisms and OSAS. However, this was not possible due to the limited quantity of data available.

Although race is a major factor in the distribution of gene polymorphisms, and our study data were from Japan [12], China [10], [13], [14], [19], [20], Turkey [9], and Brazil [11], we found no distribution differences in the gene polymorphisms evaluated. We could not conduct a haplotype analysis in this study because we could not collect original haplotype data. For the 5-HTR2A 1438G/A and STin2VNTR meta-analyses, heterogeneity existed among the studies included, which, together with the analysis of the genotype distribution of 5-HTR 2A and 5-HTT gene polymorphisms according to gender, suggests that the heterogeneity may have been caused by gender imbalances in the different studies.

During the preparation of this manuscript, a systematic review and meta-analysis of the association between 5-HT2A receptor polymorphisms and risk of OSAS was published [30]. The differences between this report and our study are as follows: our study included not only 5-HTR gene polymorphisms but also 5HTTLPR and STin2VNTR gene polymorphisms, and our study first demonstrated that gender was an important cofactor for 1438G/A allele distribution; the AA genotype increased the OSAS risk with an OR of 4.21 (95% CI: 2.83–6.25) in a recessive model in male OSAS patients, but no significant association was found in females.

5-HT is associated with circadian rhythm and breathing regulation, and the 5-HTR2A and 5-HTR2C subtypes play an important role in the maintenance of upper airway stability and normal breathing in obesity [31]. 5-HTT plays an important role in serotoninergic transmission. Thus, studies concerning polymorphisms in the 5-HTR2A and 5-HTT gene would seem to be important for determining the genetic basis of OSAS. In this study, 5-HT2A 1438G/A, the long 5-HTTLPR, and the 10 tandem repeat variant of STin2VNTR were demonstrated to be risk alleles for OSAS. Further evidence from large-scale population studies and bio-functional assessments of these alleles are essential to confirm the associations between the genotypes and phenotypes.

This meta-analysis had a number of limitations. First, the pooled subjects in our meta-analysis were relatively small. Second, some studies with negative results may not have been reported, which will likely increase the publication bias in a meta-analysis. Despite these limitations, meta-analysis is a cost-effective and reasonable approach to evaluating sporadic, inconsistent, and small-sample-size studies.

In conclusion, we demonstrated by a meta-analysis that the 5-HTR2A 1438G/A, 5-HTTLPR, and STin2VNTR polymorphisms confer susceptibility to OSAS. The AA genotype increased the OSAS risk with an OR of 4.21 (95% CI: 2.83–6.25) in a recessive model in male OSAS patients, although no significant association was found in females. Further studies using larger samples, of better designs, and that include additional ethnicities are warranted. Additionally, the biological mechanism(s) and function(s) of the 5-HTR and 5-HTT genotypes and haplotypes in the pathogenesis of OSAS should be investigated further.

## Supporting Information

Table S1 **Characteristics summary of publications included in meta-analysis.**
(XLS)

Checklist S1 **PRISMA Checklist.**
(DOC)
